# Solitary peliosis hepatis mimics a liver metastasis on contrast-enhanced ultrasound

**DOI:** 10.1016/j.radcr.2023.02.047

**Published:** 2023-03-20

**Authors:** Paul Spiesecke, Stefan Pahl, Thomas Fischer, Markus Herbert Lerchbaumer

**Affiliations:** a Department of Radiology, Charité—Universitätsmedizin Berlin, corporate member of Freie Universität Berlin, Humbold, Universität zu Berlin, and Berlin Institute of Health,Charitéplatz 1, 10117 Berlin, Germany; b Department of Pathology, Charité—Universitätsmedizin Berlin, corporate member of Freie Universität Berlin, Humbold, Universität zu Berlin, and Berlin Institute of Health, Charitéplatz 1, 10117 Berlin, Germany

**Keywords:** Peliosis hepatis, CEUS, Contrast-enhanced ultrasound, Metastasis, Ultrasound

## Abstract

Peliosis hepatis remains a rare focal liver lesion with inconclusive imaging features. The unknown pathogenesis represents a wide possible range of etiologies including the breakdown of the sinusoidal borders, a potential hepatic outflow obstruction or dilatation of the central vein of a hepatic lobule. In histopathology, a blood-filled cystlike appearance with sinusoidal dilatation was reported. On ultrasound, B-mode features are not specific demonstrating a irregular, moreover hypoechogenic focal liver lesions. Postcontrast imaging features on Contrast-Enhanced-Ultrasound may mimic a malignant lesion with irregular contrast inflow and washout during late phase. Our case demonstrates a peliosis hepatis with malignant image features on contrast-enhanced ultrasound, ruled out by PET-CT and core needle biopsy with corresponding histopathological workup.

## Introduction

Peliosis hepatis is known as a rare disease showing multiple blood-filled parenchymal cysts in parenchymal organs, which may be associated with a wide range of conditions varying from medication or infectious diseases and autoimmune diseases [1,2]. Although the pathogenesis remains unclear, histopathological postsinusoidal obstruction were hypothesized to be a potential mechanism [1]. According to current literature, only a small number of case reports and a study including 24 patients [3] demonstrated perfusion pattern on contrast-enhanced ultrasound (CEUS). Thus, we are demonstrating a case with peliosis hepatis mimicking a liver metastasis in a patient during late follow-up of a malignant melanoma.

## Case description

We demonstrate the case of a 79-year old female patient with conjunctival melanoma diagnosed in 2001. The patient was referred to the Department of Cardiology due to dyspnea under cardiac decompensation and subacute pulmonary embolism. Afterwards, the patient was referred to head and neck surgery to further perform imaging for tumor staging. Magnetic resonance imaging was avoided due to an implanted pacemaker.

**Fig. 1:**
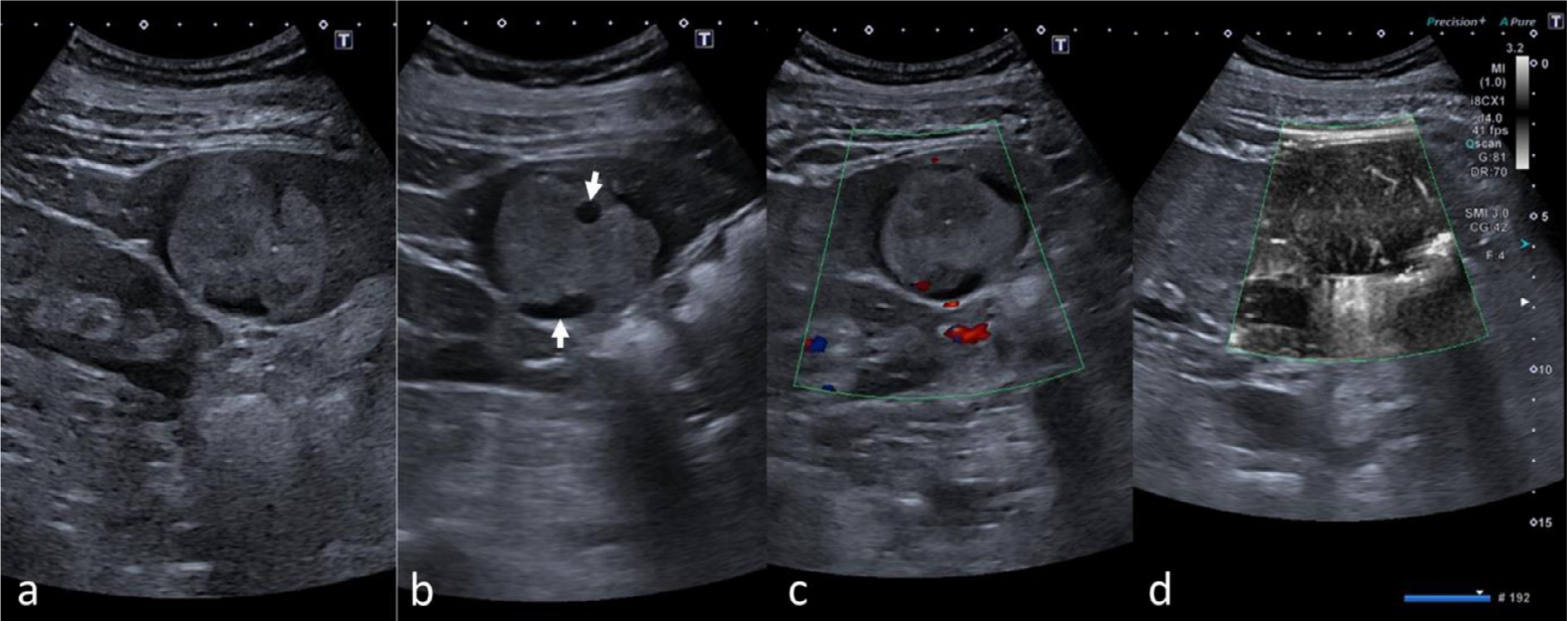
B-Mode and Doppler imaging. Peliosis hepatis demonstrating as a hyperechogenic solitary lesion (A) with hypoechogenic halo and cystic compartiments (B, arrows). On color-coded Doppler sonography, no macrovascularization was noted (C), but microvascular imaging showed small, low-flow vessels inside the lesion.

Positron emission tomography / computed tomography (PET/CT) revealed a 4 × 3 cm mass in the so-called Riedel-lobe, which is described as a normally absent, tongue-shaped process at the anterior margin of the right liver lobe overhanging the right kidney. The focal liver lesion (FLL) was slightly hypodense compared to the surrounding liver tissue on nonenhanced CT (Hounsfield units: 39 HU vs 59 HU in the surrounding liver tissue of the right lobe) and showed no sufficient contrast enhancement (HU values 53 vs 136 in the right liver lobe) after injection of 80 ml iodine-based contrast agent (Figs. 1A and B). Moreover, no PET-avidity was noted in the 18-FDG PET scan (Fig. 1C).

**Fig. 2:**
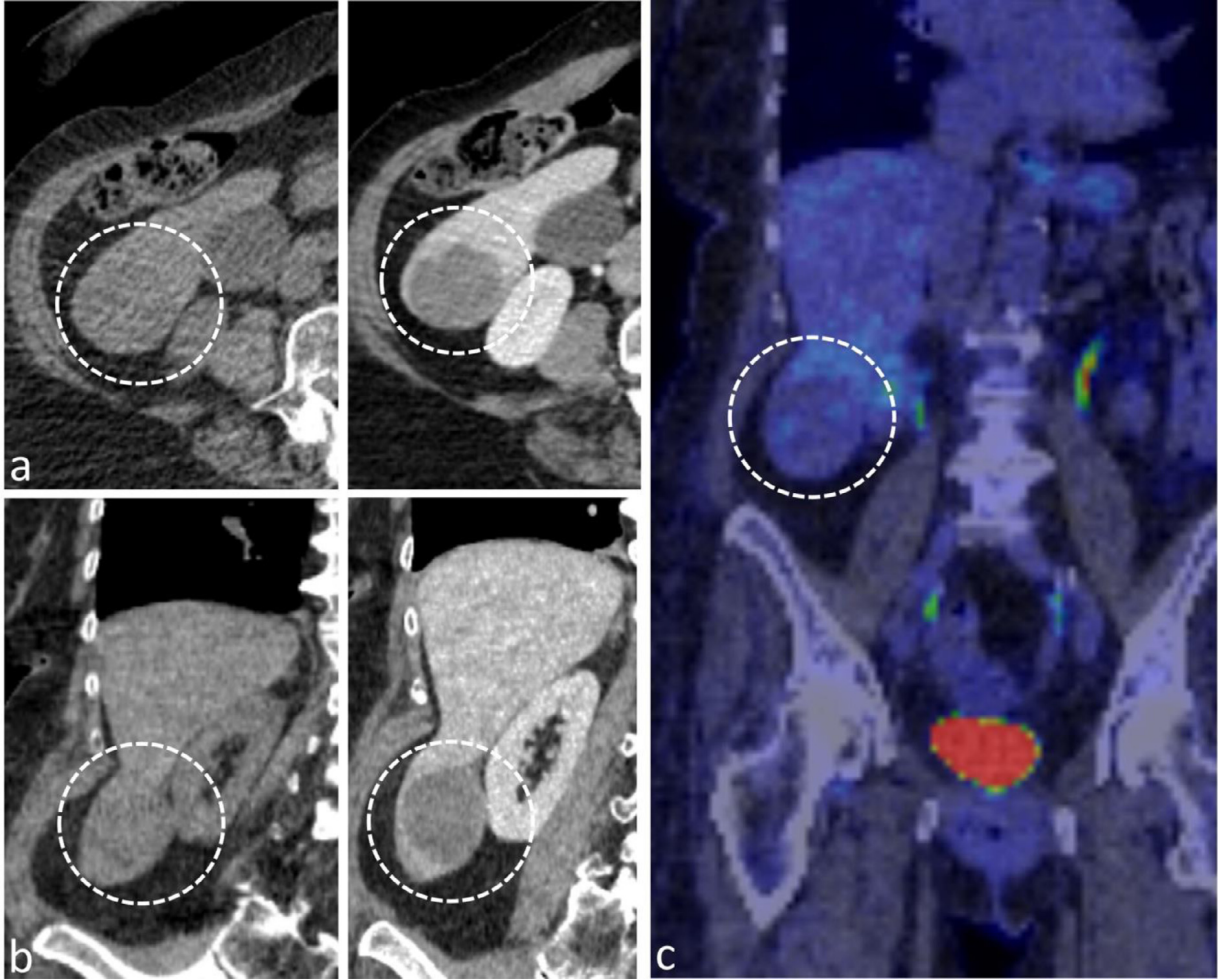
Contrast-enhanced CT and PET/CT scan. Solitary hypodense lesion without significant contrast enhancement compared to the surrounding liver parenchyma in transversal (A) and coronal plane (B) demonstrated in nonenhanced images (left) and contrast-enhanced images (right). PET/CT showed no avidity of the lesion (C).

**Fig. 3:**
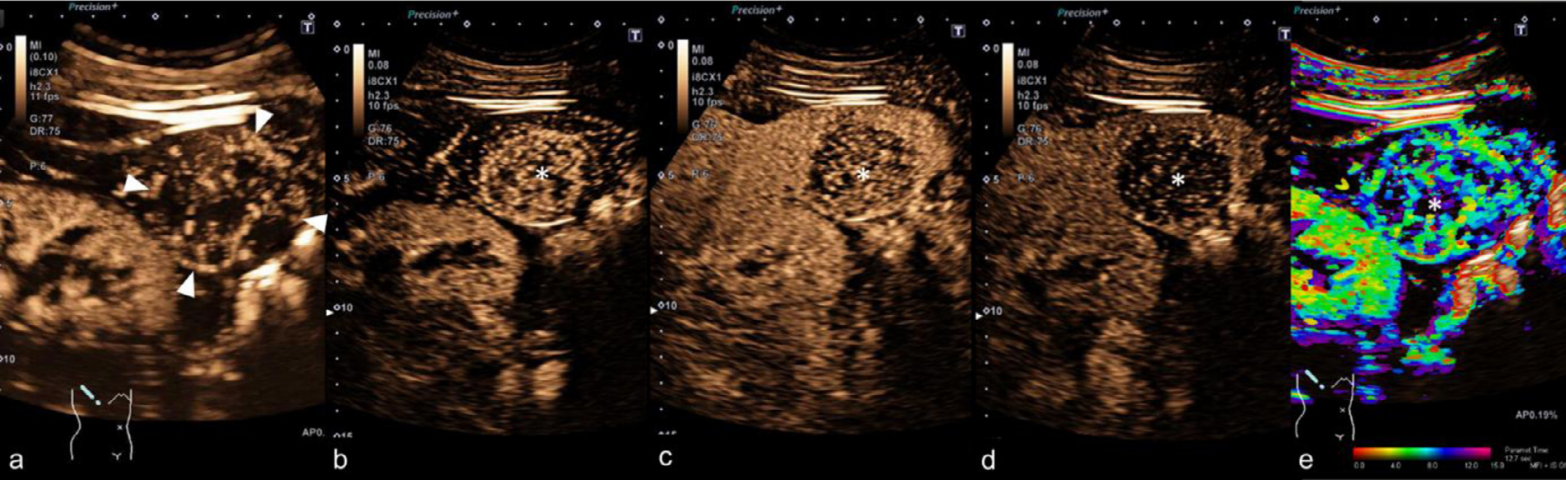
CEUS. Dynamic real time CEUS showed early irregular contrast enhancement (after 13 seconds [A], and 23 seconds [B]) with washout during portal-venous contrast phase (43 seconds, C) and late phase (2:21 minutes, D). Color-coded parametric arrival time imaging clearly depicted vessel structure with small peripheral vessels (green color, E).

To further characterize the FLL, CEUS was performed by an expert in the field with more than 20 years of experience in this field (EFSUMB Level III). The ultrasound (US) protocol using a high-end US system with 1–6 MHz convex array transducer (Aplio i800, Canon Medical Systems Corporation, Tochigi, Japan) included color-coded duplex sonography (CCDS) and superb microvascular imaging (SMI) to determine vessel structure. Afterwards, the liver lesion was examined in modified longitudinal and transversal planes with an up-to-date CEUS-specific protocol available at the time of the examination. On B-Mode US, the lesion was inhomogenous and hyperechogenic compared to the surrounding liver tissue (Figs. 2A and B). No macrovascularization was noted on CCDS (Fig. 2C), while small vessels inside the lesion were depicted on sensitive microvascular imaging mode only (eg, SMI, Fig. 2D). After bolus injection of 1.6 ml of a second-generation US contrast agent followed by a flush of 10 mL 0.9% saline solution, the FLL showed strong contrast enhancement in early arterial phase prior the surrounding liver tissue with moreover irregular enhancement pattern (Figs. 3A and B). Lesion washout was depicted 40 seconds after contrast injection followed by progressive washout over 3 minutes (Figs. 3C and D).

**Fig. 4:**
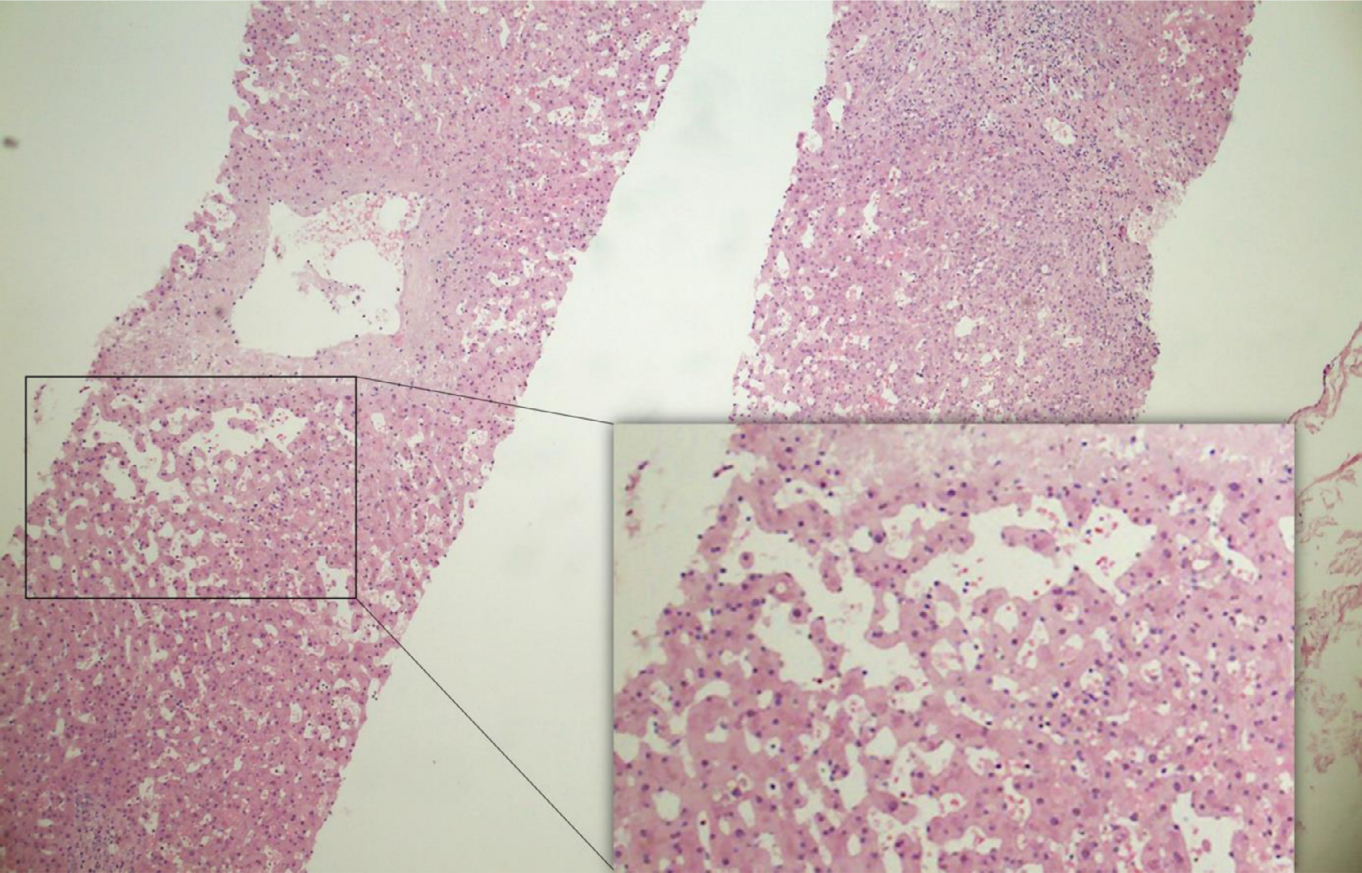
Histology. Histological section of liver biopsy specimen showing sinusoidal dilatation with widened portal fields. HE, haematoxylin-eosin stain.

A percutaneous US-guided core biopsy (16 Gauge needle) was performed due to the discordance between CEUS findings and PET/CT findings after an internal discussion of several disciplines. The histopathological report revealed no atypical cells suspicious for malignancy. The hepatic parenchymal specimen showed peliosis-like dilatation of the sinusoids and edematous widened portal fields with a mixed cell inflammatory (Fig. 4).

## Discussion

Peliosis hepatis is known as a rare disease with several underlying conditions, which is a challenge for diagnosis on established imaging modalities. Our case report shows a patient with suspicion of malignancy receiving a PET/CT scan and CEUS with discrepant findings on both modalities.

Perfusion pattern of peliosis hepatis in CEUS are quite variable including a variety of B-mode findings with hyper-, iso- and hypoechogenicity [3,4]. While the solitary FLL showed no PET avidity, CEUS clearly identified suspicious contrast enhancement pattern in form of early, irregular arterial inflow without following a typical enhancement pattern known for haemangioma or focal nodular hyperplasia. Moreover, contrast enhancement of the lesion excluded a hemorrhagic cyst, which normally shows no intraluminal enhancement.

In comparison to the results of the study by Dong et al., the lesion we present is in congruence with the described arterial hyperenhancement—but our case showed early progressive washout, whereas the authors concluded washout in the very late portal venous phase (after 1 minute) to be characteristic in peliosis hepatis [3]. Furthermore, 83.3% of the included lesions were described to be heterogeneously hypoechoic, whereas the case we present had hyperechoic appearance in B-mode US [3].

Several contrast pattern findings for peliosis hepatis are known through the literature, including centripetal contrast enhancement mimicking hemangiomas or atypical enhancement patterns not suggestible for any typical FLL [4,5]. In our case, the FLL showed irregular and strong enhancement on early arterial phase prior compared to the surrounding liver tissue. Moreover, washout was depicted after 40 seconds with progressive washout over 2 minutes, which is a well-known sign of liver metastases or cholangiocellular carcinoma [6]. Irregular enhancement pattern and slightly washout may be also present in hepatocellular adenomas. The findings of our case are in accordance to some studies, but contrarily to findings demonstrating centripetal filling without washout [7]. While several contrast patterns are descripted, our case reports may improve the knowledge of contrast pattern of this rare benign hepatic disorde.

Catchword phrases:

• Peliosis hepatis showed strong enhancement with irregular enhancement pattern
• Early washout starting after 40 seconds post injection was present
• CCDS depicted no macrovascularization, while microvascular imaging revealed some tiny vessels

## Patient consent

Our patient demonstrated in the case report signed a written informed consent for publication/research purpose.
